# Epochal changes in the association between malaria epidemics and El Niño in Sri Lanka

**DOI:** 10.1186/1475-2875-7-140

**Published:** 2008-07-24

**Authors:** Lareef Zubair, Gawrie N Galappaththy, Hyemin Yang, Janaki Chandimala, Zeenas Yahiya, Priyanie Amerasinghe, Neil Ward, Stephen J Connor

**Affiliations:** 1 International Research Institute for Climate and Society, Columbia University, New York, USA; 2 Anti-Malaria Campaign, Ministry of Health, Colombo, Sri Lanka; 3 Columbia University, New York, USA; 4 Foundation for Environment, Climate and Technology, Digana Village, Sri Lanka; 5 International Water Management Institute, Hyderabad, India

## Abstract

**Background:**

El Niño events were suggested as a potential predictor for malaria epidemics in Sri Lanka based on the coincidence of nine out of 16 epidemics with El Niño events from 1870 to 1945. Here the potential for the use of El Niño predictions to anticipate epidemics was examined using enhanced climatic and epidemiological data from 1870 to 2000.

**Methods:**

The epidemics start years were identified by the National Malaria Control Programme and verified against epidemiological records for consistency. Monthly average rainfall climatologies were estimated for epidemic and non-epidemic years; as well El Niño, Neutral and La Niña climatic phases. The relationship between El Niño indices and epidemics was examined to identify 'epochs' of consistent association. The statistical significance of the association between El Niño and epidemics for different epochs was characterized. The changes in the rainfall-El Niño relationships over the decade were examined using running windowed correlations. The anomalies in rainfall climatology during El Niño events for different epochs were compared.

**Results:**

The relationship between El Niño and epidemics from 1870 to 1927 was confirmed. The anomalies in monthly average rainfall during El Niño events resembled the anomalies in monthly average rainfall during epidemics during this period. However, the relationship between El Niño and epidemics broke down from 1928 to 1980. Of the three epidemics in these six decades, only one coincided with an El Niño. Not only did this relationship breakdown but epidemics were more likely to occur in periods with a La Niña tendency. After 1980, three of four epidemics coincided with El Niño.

**Conclusion:**

The breakdown of the association between El Niño and epidemics after 1928 is likely due to an epochal change in the El Niño-rainfall relationship in Sri Lanka around the 1930's. It is unlikely that this breakdown is due to the insecticide spraying programme that began in 1945 since the breakdown started in 1928. Nor does it explain the occurrence of epidemics during La Niña phase from 1928 to 1980. Although there has been renewed coincidence with El Niño after 1980, this record is too short for establishing a reliable relationship.

## Background

While many factors play a role in the distribution of malaria and occurrence of malaria epidemics; climate is considered a major determinant [1-3]. Temperature, rainfall, and humidity affect breeding and survival of vector mosquitoes and development of malaria parasites within the mosquitoes. Many epidemics have occurred during droughts as river margins retreat leaving numerous pools suitable for vector breeding, or in the season following a drought when rains return to normal and breeding sites form on depressions in the dried up river beds and small reservoirs and river margins [4]. This post-drought epidemic scenario often poses a major public health problem among populations whose vulnerability may have been heightened due to a period of poor nutrition and lowered immunity [5-8].

A major constraint to a more focused approach to malaria control in many epidemic prone regions is the lack of a prediction system that makes use of climatic information. The WHO advocates the use of such warning systems [7,9] and it is a priority for malaria control in Sri Lanka [10]. It is in this context that this paper re-examines the relationship of ocean-atmosphere phenomenon of El Niño-Southern Oscillation (ENSO) with epidemics [11] in Sri Lanka.

El Niño is a shift in the pattern of oceanic warming and atmospheric circulation centred in the Pacific Ocean that recurs typically every few years. It is associated with anomalously warm sea surface temperatures (warm phase) in the equatorial Eastern Pacific. Its cold phase analogue is referred to as La Niña. The atmospheric component of this climatic phenomenon has been referred to as the Southern Oscillation, and collectively the ocean-atmosphere phenomenon is referred to as El Niño/Southern Oscillation (ENSO). Rainfall is often enhanced in Sri Lanka during El Niño events from October to December and diminished from July to August and January to March [12]. Any relationships between ENSO and malaria risk could be useful as ENSO forecasts are routinely available [13].

El Niño was suggested, by Bouma and Van Der Kaay [14], as a potential early warning indicator for epidemics in Sri Lanka, as nine out of sixteen epidemics coincided with El Niño years from 1870 to 1945. Of twenty El Niño years in the same period, nine years coincided with epidemic episodes. They also reported that the El Niño episodes led to rainfall anomalies during the North-East monsoon period in the "Dry Zone" that may be related to malaria epidemics. Note: that their usage of monsoon duration (North-East monsoon lasts from October to December and South-West monsoon lasts from March to July) is at variance with the usage in the meteorological literature (North-east monsoon from December to February and South-West Monsoon from May to September) [12]. They found that there was a significant influence of the October to December rainfall on peak malaria transmission seasons in the middle and end of the year.

The relationship of rainfall patterns and malaria incidence has been previously investigated. For example, an analysis in eight districts from 1901 to 1934 found that the decrease in rainfall during January to May was related to epidemics in the boreal "summer", and the decrease in June to September rainfall was related to epidemics in the "winter" [15]. A weak relationship was established between rainfall and malaria incidence in North-Central region from 1979 and 1995 [16].

While many factors such as housing types and distance to water bodies need to be considered in assessing malaria risk in Sri Lanka [17], there is a need for understanding geographic, seasonal and inter-annual specificity in impending malaria risk. Such spatio-temporal identification could be particularly valuable in communicating with resource managers who can use water and land management techniques to reduce mosquito breeding in pools in river beds [18]. Recent declines in malaria incidence in Sri Lanka have provided an incentive to develop a malaria early warning system (MEWS). Since resources for epidemiological monitoring and surveillance is bound to decline following control success (this has happened many times in Sri Lanka's history), leaving populations with reduced immunity there is the risk of major resurgences [19]. In this context, using routinely collected climate, hydrological and environmental data for MEWS becomes more important.

In this paper, the association between El Niño and epidemics is re-examined using enhanced climatic and epidemiological data from 1870 to 2000. On finding that the association held up from 1870–1928 and possibly after 1980, the relationship between rainfall and ENSO was investigated to identify causes for the epochal nature of this association.

### Geography and climate

**Figure 1:**
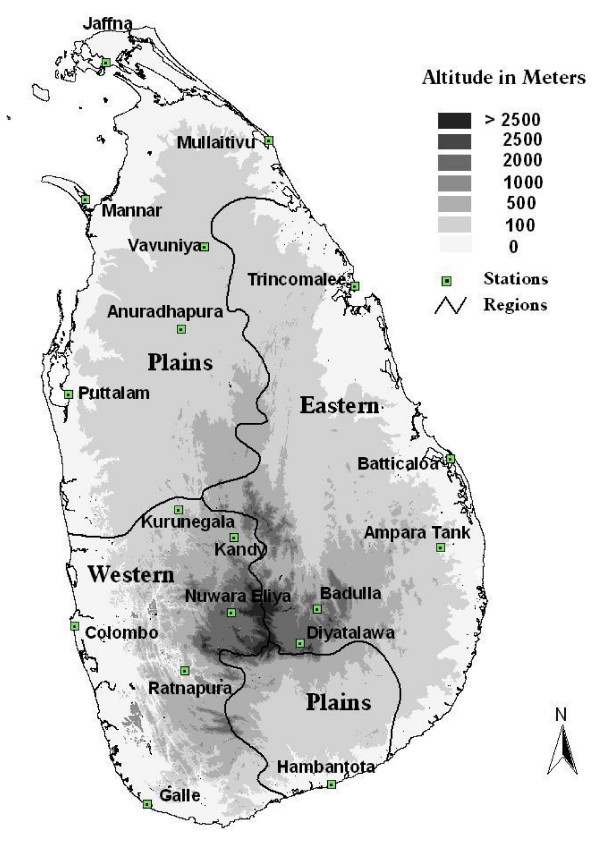
**Topography of Sri Lanka is shown as shading along with locations of meteorological observatories**. The homogeneous climate regions Eastern, Western and Northern and Southern Plains are also shown.

The island is 400 km in the North-South extent and 250 Km in the East-West extent with a mountain massif located in the south-central region which rises to 2.5 Km (Figure 1). The climate in most of Sri Lanka is suitable for malaria transmission. The mean annual temperature is 27°C with a diurnal range of 6°C on average. Rainfall ranges from 500 to 5,500 mm annually, and relative humidity ranges are above 65%, except in a few regions.

The mean annual cycle of rainfall in Sri Lanka is bimodal with a major mode from October to December, and a subsidiary mode from April to June. The rainfall peaks coincide with the passage of the "Tropical Convergence Zone" over the island around May and October. Rainfall during these seasons is high through most of the island. During other periods there is regional variability primarily due to orographic and cyclonic influences. Storms and cyclones affect the rainfall along the north-eastern coast from November to January. The rainfall in the Western region is enhanced from April to October due to topographically enhanced rainfall on the windward side of this mountain ridge, and enhanced on the Eastern side from November to February. ENSO and Indian Ocean sea surfaces are the major factors that influence the inter-annual variability of Sri Lanka's climate system.

### History of epidemics

**Figure 2:**
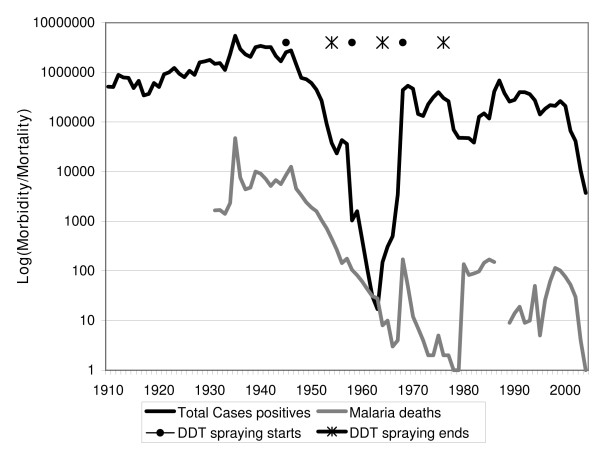
**Annual island-wide morbidity and mortality due to Malaria for 1910 to 2003 is shown on a logarithmic scale**. These are annual estimates from the Anti-Malaria Campaign of Sri Lanka and its predecessors.

The long history (Figure 2) of malaria control in Sri Lanka [20,21] offers a number of lessons for current malaria control services both in Sri Lanka and elsewhere. From 1868 to 1948 there have been 20 epidemics in Sri Lanka with case burdens reaching three million (60% of population) in the 1934–35 epidemic [22]. Sri Lanka was remarkably successful during the "Global Malaria Eradication Campaign" reducing its burden from a typical two million cases per year in 1948 to just 17 cases in 1963 [23,24]. These control efforts included DDT-spraying [24] and environmental measures such as anti-larval flushing of rivers [25,26]. It appears that relaxation of control efforts around this time led to rapid and dramatic resurgence reaching 537,700 registered cases in 1969. Following this major epidemic, malaria control was revitalized and case numbers were brought down markedly, but another epidemic in 1975 produced 400,700 cases. Thereafter, there have been epidemics in 1983 (127,000), 1987 (680,000 cases), 1991–2 (400,000 cases) and 1999 (290,000 cases). Presently Sri Lanka hosts 20 million people in a land area of 65,000 square kilometres (Figure 1). *Plasmodium falciparum*, which historically has been of low prevalence in comparison to *Plasmodium vivax*, has increased from 5% to above 25% of cases over the past few decades and is increasingly becoming resistant to the first-line anti-malarial drug, chloroquine [27], [28]. Recently malaria incidence has declined as Sri Lanka's Anti-Malaria Campaign (AMC) has been implementing more effective malaria control activities. Since 2000 the incidence has reduced even further (Figure 2).

Epidemics occur at three to five year intervals coinciding with low rainfall and high temperature [15], which favors the propagation of the vector *Anopheles culicifacies *sustained by secondary vectors such as *Anopheles subpictus and Anopheles annularis *[29,30]. Malaria transmission in Sri Lanka is complex and vector breeding occurs in a range of settings, including: riverbeds, gem mining pits, seepage areas from irrigation reservoirs and canals and animal foot prints in moist regions, all with different ecological dynamics. The development of epidemic foci has been associated with slash and burn cultivation in poorly accessible deep forested areas, abandoned pits from gem mining areas [31] and the migration of non-immune populations to endemic areas under resettlement programmes. These conditions occurred in late 1967 and 1968 prior to the 1969 epidemic.

## Methods

### Mortality and morbidity data

Annual mortality and morbidity data from 1911 and monthly incidence data from 1961 up to 2003 were obtained from the Anti-Malaria Campaign (AMC), the publications of its predecessors and compiled from archival records.

### Identification of epidemics

An epidemic is the occurrence of cases in excess of the number expected in a given place and time [32,33]. Thus, in non-endemic areas any transmission including a few cases could constitute an epidemic.

The difficulties in using this definition for endemic areas include not knowing the "expected" number of cases. Endemic malaria has cycles of several years which is usually determined by climate and amplified by loss of immunity in periods of low transmission.

Several practical epidemic detection algorithms have been proposed for monthly case data [6]. These detection algorithms rely, for example, on increases from the mean by 1–2 standard deviations or prevalence above some thresholds such as the highest quartile. All these methodologies have some shortcomings arising from the imprecision in definition of epidemics and limitations in applicability due the limited availability of data.

Malaria epidemic years before 1900 have been identified in reports of Ceylon's Civil Medical Department [20]. The Director of Medical and Sanitary Services was mandated to undertake malaria control in the first half of the 20^th ^century. These functions were carried out by the Anti-Malaria Campaign in the second half of the 20^th ^century. These services have undertaken identification of epidemics as an "expert opinion". Vital statistics, which included morbidity, mortality, birth rate and infant mortality rate were used to identify epidemic years between 1901 and 1934 [15]. The dramatic increase of death and infant mortality rates with a conspicuous fall of the birth rate marked the epidemic years. The collection of blood films for *P. falciparum *and *P. vivax*, and total malaria parasite positives can be analysed to identify epidemic years apart from non-epidemic years [34]. In previous analysis on El Niño and epidemics, Bouma and Van der Kaay [14] used the identification from the Director of Medical and Sanitary Services [15,20] which was confirmed by retrospective review in 1935 [22]. The epidemic years were identified as 1877, 1880, 1884, 1891, 1906, 1911, 1914, 1919, 1923, 1928/29, 1934/35, 1939/40, 1943 and 1945/46. This list was used in the analysis reported here to facilitate comparison until 1945. The epidemics since 1945 were identified as 1967/69, 1975, 1983, 1986–1988, 1990/92 and 1999/2000 [15] by the Anti-Malaria Campaign of Sri Lanka.

### Rainfall

The monthly rainfall data for 17 main meteorological stations from January 1869 to December 2003 were obtained from the Sri Lanka Department of Meteorology (Figure 1). An island-wide rainfall index was constructed based on the records averaged over the 17 well-distributed stations and diagnostics were reported [12]. This rainfall index was used for further analysis here.

### ENSO data

The ENSO state may be represented by the anomalies of sea-surface temperature (SST) from the seasonal average [35]. The mean SST anomalies over the eastern equatorial Pacific (5°N – 5°S, 170° – 120°W) are widely used as an ENSO index (NINO 3.4) [36]. An "El Niño" is designated when six consecutive five-month-running-averages of SST's stayed above 0.4°C. A "La Niña year" is designated when six consecutive five-month-running-averages of SST's stayed below 0.4°C.

ENSO states had been identified by calendar year in some of the early literature. For example, Quinn *et al *[37] provided a listing by year of El Niño events and a measure of event intensity (very strong, strong, moderate, weak, and very weak) beginning in 1726. The measures used to define the El Niño and its intensity was primarily based on phenomena along the coast of South America, and was often qualitative. Note that the focus of Quinn *et al *was on the Southern Hemisphere summer and fall – which refers to the end of the year. Bouma and Van Der Kaay [14] defined the El Niño years following Quinn *et al *[37] with additions to the record from Rasmussen and Carpenter [38]. They take the first year of a multi-year event as significant.

The definition of ENSO used in this analysis is based on current practice which is based on the use of SST estimates going back to 1856 [36].

### Composite analysis

The monthly average rainfall that characteristically prevails during each ENSO phase may be estimated by averaging the rainfall during each of these phases. For example, to estimate the average monthly rainfall during the El Niño phase, the intervals during which the El Niño phase prevails is identified (based on NINO3.4 > 0.4°C). Thereafter, the monthly rainfall values during these intervals alone are used to estimate the average monthly rainfall. Similarly the average monthly rainfall during the Neutral and La Niña phases are estimated based on the rainfall data when the Neutral (-0.4 < NINO3.4 < 0.4 °C) and La Niña (NINO3.4 < -0.4°C) phases prevailed.

The standard error may be estimated as (*s*/√*n*) where *s *is the sample standard deviation and *n *is the number of samples.

### Hypothesis testing, sensitivity and specificity

The hypothesis of positive association of epidemics with El Niño is tested with chi-square (χ^2^) test for its statistical significance.

In a binary classification system, such as the use of El Niño to identify epidemics, sensitivity and specificity are one approach to quantifying the ability of one variable (such as El Niño) to predict the other variable [39]. The sensitivity identifies the proportion of true positives that are identified as such by the predictor. The specificity measures the proportion of negatives, which are identified as such.

### Windowed correlation analysis

Correlation analysis was used to identify relationships between the monthly ENSO indices and rainfall using Pearson algorithm [40]. The evolution of correlations between variables was captured with windowed running correlations. To assess the sensitivity of the running correlations of various window lengths can be considered considered.

## Results

The following issues are addressed below: (a) identification of the annual cycle of malaria incidence, (b) identification of ENSO influence on rainfall, (c) objective identification of epidemics, (d) characterization of the relationships between El Niño and epidemics, (e) identification of the rainfall anomalies during epidemics and different phases of ENSO, and (f) characterization of the relationship between epidemics, ENSO and rainfall focusing on decadal changes.

### Annual cycle of malaria incidence

**Figure 3:**
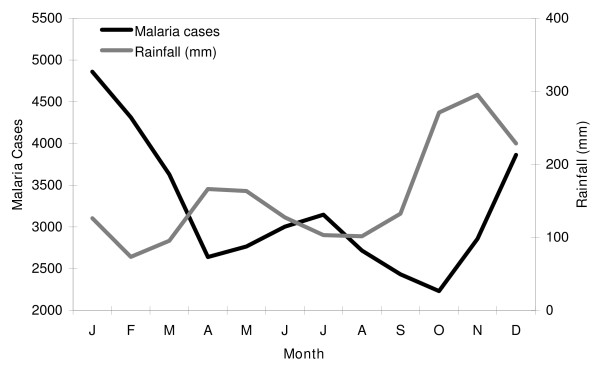
**The monthly average incidence of malaria in Sri Lanka for 1961–2000 compared with the monthly average rainfall for the same period**. Malaria incidence peaks in January and July. Rainfall peaks in November and May.

Monthly average morbidity due to malaria was estimated from incidence data from 1960 to 2003 (Figure 3). The incidence of malaria is bimodal with peaks in December-January and in June-July. The peak at the end of the year is more pronounced than that during the middle of the year. The peaks are two months after the peak in rainfall in November and May. This bimodal distribution of malaria seasonality is retained in the 25 districts. The December-January peak dominates in the Northern and Eastern half of the country.

**Figure 4:**
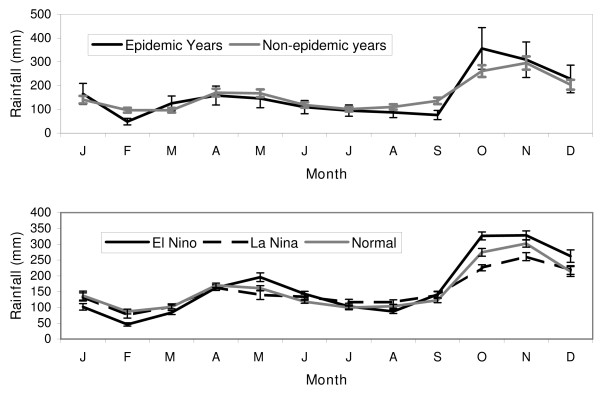
**(a) Monthly average rainfall climatology for epidemic start years (1877, 1880, 1884, 1891, 1906, 1911, 1914, 1919, 1923, 1928, 1934, 1939, 1943, 1945, 1967, 1986, 1990) and for non-epidemic years for the period of 1876 to 2000**. (b) Average monthly rainfall, and that during El Niño and La Niña phases. The error bars represent the standard error of the estimate.

The features that stand out in comparing the climatology during epidemic and non-epidemic years (figure 4a) are a drop in rainfall, during February and September and an increase in rainfall during October.

### ENSO influences on rainfall

The composite climatology for El Niño, Neutral and La Niña was constructed (Figure 4b). Generally, during an El Niño event, rainfall increases from October to December and May, and decreases during January to April and July to August [12]. The influence of La Niña events is opposite that of El Niño during October to December (decreases) and July to August (increases), but not during January to March (decreases) El Niño and La Niña episodes usually begin in late boreal spring or fall and last for a period ranging up to a couple of years. Note that the modulations of rainfall during El Niño episodes (Figure 4b) and during epidemics (Figure 4a) are similar over the entire record. This similarity supports the existence of a relationship between El Niño and epidemics.

### Objective Identification of Epidemics

As an exercise in validation of the contemporaneous expert assessment of epidemics by the government health authorities, the available historical morbidity data may be used in an objective scheme to identify epidemics. The longest morbidity data available to us was annual incidence data aggregated for the island from 1910. Epidemics that are pronounced during some months of the year and in some regions are less prominent when annual aggregated national data is considered rather than monthly district-wise data. Thus a scheme based on consolidated annual data may miss out epidemics that was localized in a few months or in a region but should generally pick out the significant epidemics.

A methodology that takes account of the large decadal variation in morbidity in Sri Lanka (Figure 3) is needed and the simplest of these methods identifies unusual rise in a short window. Here, occasions in which the annual incidence exceeded that of the five-year running mean by half of the standard deviation were identified as an "epidemic". This threshold was arrived at empirically as one that was both simple and that would capture a number of epidemics comparable to the expert opinion. The use of short windows has its shortcomings due to possible rapid variations in mean and standard deviations from one window to the next. Allowance also has to be made for the fact that the epidemics often occur across the calendar year (i.e. from November to February – Figure 3). Thus there can be instances where the objective scheme may identify the adjacent year as an epidemic.

The objective scheme picked out 13 out of the 14 epidemics as identified by expert opinion since 1912. The exception was 1919 but this is a year with a relatively high case load. This was an instance which did not reach the requisite threshold due to a high standard deviation. There was another case that were identified as epidemics by the objective scheme (1956/57) which were not identified as so by expert opinion. This case was during the eradication period with low case-loads (144 cases). Thus the expert determination by the Sri Lanka health authorities is well supported by an objective test for epidemics since 1910. The official assessment was used as it provides a longer record of epidemics starting in 1870.

### El Niño and epidemics – statistics

The association of epidemics with El Niño held from 1870 to 1927 at statistically significant levels (Table 1a). The probability of an epidemic during El Niño phase was 44% (seven epidemics from 16 El Niño) compared to 5% (two in 42) during other years (χ^2 ^= 14.4, p < 0.001). The five epidemics between 1928 and 1945 did not coincide with El Niño events. Only four out of 11 epidemics from 1928 to 2000 (Table 1b) occurred during El Niño events. From 1928–1980, only one of seven epidemics occurred during El Niño episodes (Table 1c). Three out of four epidemics from 1981 to 2000 coincided with El Niño events (Table 1d) but the number of events are too few for reliable statistics.

**Table 1:** Epidemic start years in Sri Lanka in relation to prevailing ENSO phases between: (a) 1870 and 1927. (b) 1928 and 2000. (c) 1928 and 1980 and (d) 1981 and 2000.

(a) 1870–1927			
	Epidemics	Non-epidemic	Total

El Niño	7	9	16
Non-Niño	2	40	42
Total	9	49	58

(b) 1928–2000			

	Epidemics	Non-epidemic	Total

El Niño	4	14	18
Non-Niño	7	58	65
Total	11	72	83

(c) 1928–1980			

	Epidemics	Non-epidemic	Total

El Niño	1	11	12
Non-Niño	6	35	41
Total	7	46	53

(d) 1981–2000			

	Epidemics	Non-epidemic	Total

El Niño	3	3	6
Non-Niño	1	23	24
Total	4	26	30

The sensitivity and specificity for 1870 to 1927 are 0.78 and 0.81 respectively. However, for the period from 1928 to 1980 the sensitivity drops to 0.14 while specificity is not overly diminished (0.76). The sensitivity picks up to 0.75 and specificity to 0.88 for the 1981 to 2000 period.

The transition from frequent El Niño-epidemic co-occurrences to infrequent co-occurrences took place around the 1930's. This was well before the more effective malaria control programmes including insecticide spraying was instituted in the mid-1940.

The climatology of rainfall during years when there were epidemics was computed (Figure 4a) along with the rainfall climatology during other years. The rainfall during epidemic years shows a drop during February, August and September and a rise during October and November as seen in the figure and previously reported [12]. On average, the rainfall anomalies during El Niño events (Figure 4b) resemble that during epidemic years (Figure 4a).

### El-Niño and epidemics

#### Inter-annual relationship

**Figure 5:**
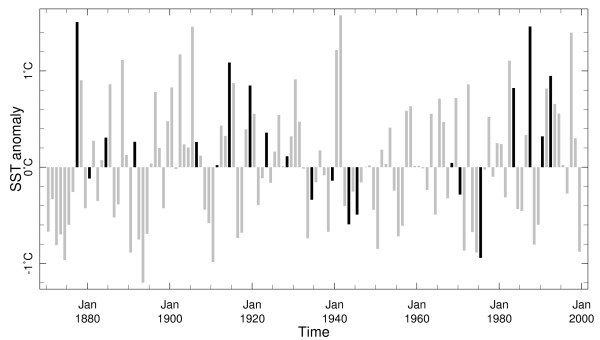
**Annual average of SST anomalies in the NINO3.4 region**. Black bars: epidemic start years. Grey bars: non-epidemic years.

This relationship may be examined in detail by representing an index of ENSO alongside epidemic start years. The inter-annual relationship between ENSO and epidemics may be brought out by representing the NINO3.4 values and epidemic start years together (Figure 5). NINO3.4 values above 0.4 are an indicator of the prevalence of an El Niño and values above 1.4 represent the strongest El Niño events. It is possible that while the average NINO3.4 value over the year is below 0.4 for the whole year that a five-month average may exceed 0.4 during that same year. Thus some caution is warranted in interpretation even when annual NINO3.4 values drop below 0.4. The NINO3.4 values range above 1.4 during the major El Niño events of 1877, 1911, 1941/42, 1982/83 and 1997. Values below -0.4 are a pointer to a La Niña event.

The epidemic years are represented with different shading (Figure 5). Figure 5 shows that until 1930, epidemics tended to coincide with El Niño events except in one case. From 1930 to 1980, epidemics have tended to occur during the periods with a La Niña tendency. After 1980, three of four epidemics coincided with El Niño episodes.

#### Epochal changes in the ENSO and rainfall relationship

Based on the shift in relationship of ENSO with rainfall as detailed above, three "epochs" may be considered (1870–1927, 1928–1980 and 1981–2000). Composites for rainfall that prevailed only during each of the El Niño, Neutral and La Niña phases for these three epochs.

**Figure 6:**
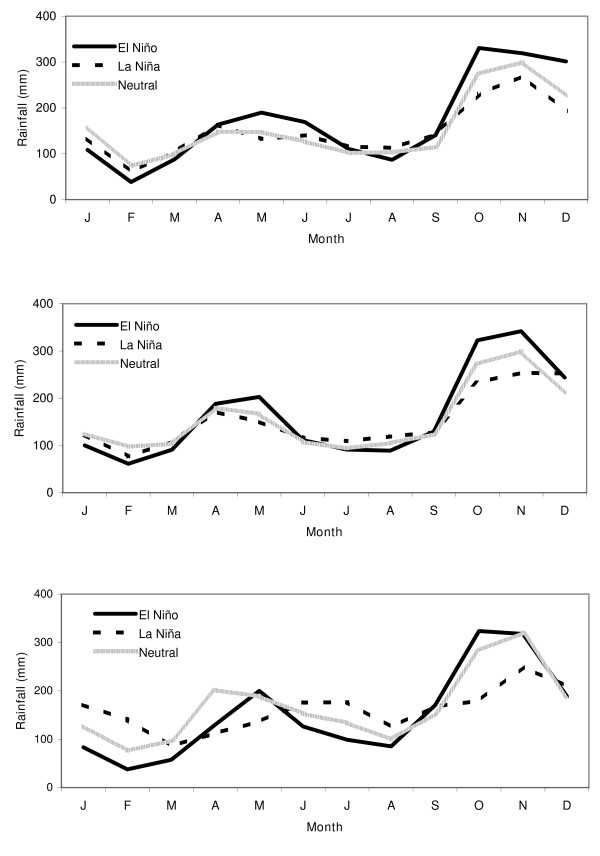
**Monthly average rainfall in Sri Lanka during El Niño, Neutral and La Niña phases from a) 1870 to 1927, b) 1928 to 1980, and c) 1981 to 2000**.

During all three periods, the rainfall during January to March has diminished during El Niño episodes and the rainfall from October to December has enhanced (Figure 6). Between 1928 and 1980, the rainfall from April to September among the different ENSO phases is similar (Figure 6b). During El Niño episodes before 1928 (Figure 6a) and after 1980 (Figure 6c), there has been an enhancement of rainfall during May and a decline in rainfall in July and August. Thus, the distinctions in the April to September rainfall during different ENSO phases go through a transition before 1928 and after 1980.

### ENSO and rainfall – windowed correlations

**Figure 7:**
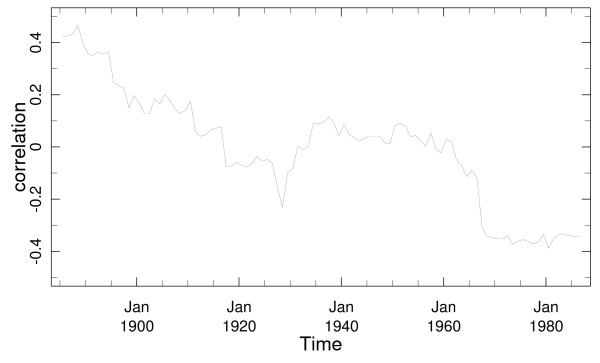
The running correlation with a 30-year window between Sri Lankan average rainfall and the ENSO index of NINO3.4. The year shown in the x-axis represents the central year of the 31 year window.

The relationship between ENSO and rainfall can be investigated further using a running correlation between the ENSO index of NINO3.4 and rainfall (Figure 7). This relationship between the October to December period has been largely consistent over the last 130 years and El Niño events have been associated with enhanced rainfall [41]. The relationship for the rest of the year (January to September) shows a substantial positive correlation (> 0.34 the 95% confidence level) in the early part of the record that subsides to near-zero after 1920's. The running correlation turns negative after 1970 reaching magnitudes that are significant at the 95% level. This drop is robust with respect to window-size and is also found for the April to September season. This analysis confirms that there have been dramatic changes in the ENSO influence on Sri Lanka over the last 130 years.

The relationships (where El Niño led to enhanced rainfall) were statistically significant at the 90% level for windows that cover the period from 1869 to 1910. Similarly the inverted relationship in the latter part of the record (where El Niño leads to reduced rainfall) is statistically significant at the 90% level for windows that include the years from 1957 to 2000.

Note, that a more detailed analysis of the running windowed correlation between rainfall and ENSO indices for the Western region was previously reported [42] and this report also documents other physical evidence for decadal changes such as changes in atmospheric circulation patterns associated with ENSO and the reports of other researchers who have observed similar phenomenon across the South Asian and Indian Ocean region.

## Discussion

The use of updated definitions of El Niño confirms the association between El Niño and epidemics from 1870 to 1928 but the relationship did not sustain after 1928. Not only did this relationship breakdown but epidemics were more likely to coincide with a La Niña tendency in the subsequent four decades.

The relationship between ENSO and rainfall underwent an epochal shift in 1930's. Prior to the 1930's, the January to September and April to September rainfall increased on average during El Niño episodes. After this period, the January to September and April to September rainfall and streamflow decreased during El Niño events. This epochal shift in the relationship between ENSO and climate can explain the breakdown in salience of the relationship between El Niño and epidemics and subsequent co-occurrence (in the next four decades) with La Niña episodes.

An alternative explanation for the breakdown in the association between El Niño and epidemics is due to the enhanced malaria control programme since 1945. While there has been reduced incidence of malaria since 1945, there are no plausible explanations as to how the control programme can explain the shift in epidemic occurrence from years with El Niño tendency to that with La Niña tendency. In addition, the effective control programme started in 1945, many years after the breakdown of the El Niño epidemic association (after 1928).

After 1980, there has been renewed coincidence of the El Niño and epidemics. The rainfall composites during El Niño, Neutral and La Niña phases show similar anomalies after 1980 as was found before 1928. The record from 1981 to 2000 is short and it is not possible to obtain a statistically significant relationship. Note, that there are several changes in the relationship between climate and ENSO since the 1980's including a strengthening in the relationship between ENSO and the October to December Sri Lankan rainfall [41].

This analysis demonstrates the need for caution in the attribution of enhanced malaria risk to El Niño episodes. Such attribution should be grounded in an understanding of the decadal or epochal variability in ENSO-malaria and ENSO-rainfall relationships.

## Conclusion

The association between El Niño and epidemics from 1880 to 1927 was confirmed. Epidemics had a stronger association with the La Niña occurrences than with El Niño from 1930 to 1980. The breakdown of this association around 1928 is likely due to a recently identified epochal change in the El Niño-rainfall relationship in Sri Lanka around the 1930's rather than due to the insecticide spraying programme that began in 1945. Although there has been renewed coincidence of the El Niño after 1980, the record is too short for establishing a statistically significant relationship.

## Conflict of interests

The authors declare that they have no competing interests.

## Authors' contributions

The conception and design of the study was undertaken by LZ, NW, and SJC. The analysis and interpretation of the data was undertaken by GG, HY, JC, ZY, and PA. The drafting of this manuscript and revision of the data was undertaken by all authors. All authors have read and approved the final manuscript.
